# Convoys and Their Care

**Published:** 1919

**Authors:** 


***Convoys and their Care. The Admitting Section of an Evacuation Hospital during Combat.*** By G. de TARNOWSKY, Major U. S. Army. *The Journal of the American Medical Association*, October 19, 1918.

The author insists upon the importance of the sorting and admission of patients, and speaks of the duties of the "sorting officer" who should always be a surgeon of quick and mature judgment.

*Personnel and Duties of the Admitting Squad.* One sergeant and from 4 to 6 privates will suffice for the ordinary convoy; this number may have to be doubled or trebled according to casualties. Rapidity and accuracy are essential. The hospital organization must be made elastic enough to meet all contingencies. The patients, classed as walking cases, line up in front of the clerk's table, give up their field medical cards, receive a ward ticket and pass on to the buffet. Stretchers are laid in a row in the admitting tent and the clerks pass rapidly from one to the other recording all necessary data in duplicate, for all urgent cases. As soon as the duplicate clinical record is filled out, with the ward number recorded on it, the bearers carry each patient to the receiving ward, returning litter, pillow and blankets, to the ambulance waiting outside. A weekly list of convoys is made up with the total number received each day from 7 a. m. to 7 p. m., and vice versa. A properly organized admitting tent and competent director of surgical material will easily quadruple the output of operative team.

*Sorting (Triage) of the Wounded.* The first complete diagnosis must be made in the evacuation hospital. The admitting officer has the difficult task of deciding on the order of precedence. Theoretically and during relatively calm periods, each surgical team should examine its patients before they are admitted to the operating room; practically, this is impossible during active fighting. On the admitting officer must also devolve a diagnosis of the relative urgency of each case, its confirmation by the fluoroscope and the surgical preparation of the wound area by the ward orderlies and nurses.

*Admitting Officer and Director of Surgical Material.* Having supervised the admission of a convoy, the admission officer should devote his attention to severe cases. In order to avoid contamination by lice, etc., a sheet is placed over the blanket of the bed. The patient is undressed, cleansed, and light diet is furnished from the buffet to all patients who do not suffer from intra-abdominal lesions, concussion or coma. In very severe shock cases, the patients are not undressed at all but heat is applied, hot drinks are given, or they are transferred to special heat chambers (chambres chauffantes). Having sorted the severely wounded into urgent surgical, observation and borderline cases, the admitting officer directs the former to the X-ray room. All severe observation and borderline cases are transferred to the different surgical wards where they are again examined and booked for operation. The admitting officer, who is also director of surgical material, should keep in constant touch with the stretcher-bearers, X-ray and anesthetizing rooms, thus insuring a constant stream of wounded towards the operation room. In order to assure a better postoperative treatment of critical cases, a recovery ward is necessary; in it the surgical teams can follow their cases from day to day.

*Surgical Dispensary for Walking Wounded.* A dispensary tent proves of great value during heavy fighting. It should be equipped with sterilizer, instruments and dressings. The dispensary dresser is furnished with a daily list of walking wounded.

*The Admitting Tent Buffet.* The buffet should consist of a section of tent or barrack with tables on each side of its long axis. One table holds tea, coffee, chocolate or cocoa, kept hot by means of a Primus lamp, with a sufficient number of cups and hot water to wash them in. The opposite table should have bread and butter, sandwiches, hash, or some other hot dish. Two orderlies under the supervision of a nurse will suffice, one of them carrying trays to the stretcher patients.

*The Delousing and Bathing Barrack or Tent.* The equipment of the bathing tent or barrack will necessarily vary with the terrain and available sanitary resources. A pitcher of water, a basin and a cup of kerosene, with soap and towel, represent the essentials; shower baths with hot water are desirable whenever obtainable.

*Dimensions and Plans of Admitting Sections.* For a 2,000 bed evacuation hospital, 5 tents or barracks, approximately 6 by 18 yards, will be required. Of these, one is used for admissions, one for buffet and undressing room, 2 for stretcher cases, and one for a bathing-room.

The general plan of admission is the result of observations made along the Franco-British front in 1917-18. If carried out, the scheme will be found to be both time- and life-saving.

